# Suppressed autophagy flux in skeletal muscle of an amyotrophic lateral sclerosis mouse model during disease progression

**DOI:** 10.14814/phy2.12271

**Published:** 2015-01-19

**Authors:** Yajuan Xiao, Changling Ma, Jianxun Yi, Shaoping Wu, Guo Luo, Xiulong Xu, Pei‐Hui Lin, Jun Sun, Jingsong Zhou

**Affiliations:** Department of Molecular Biophysics and Physiology, Rush University School of Medicine, Chicago, Illinois; Department of Physiology, Kansas City University of Medicine and Biosciences, Kansas City, Missouri; Department of Biochemistry, Rush University School of Medicine, Chicago, Illinois; Department of Cell Biology, Rush University School of Medicine, Chicago, Illinois; Department of Surgery, Davis Heart and Lung Research Institute, The Ohio State University Wexner Medical Center, Columbus, Ohio

**Keywords:** Amyotrophic lateral sclerosis, cell physiology, skeletal muscle

## Abstract

Accumulation of abnormal protein inclusions is implicated in motor neuron degeneration in amyotrophic lateral sclerosis (ALS). Autophagy, an intracellular process targeting misfolded proteins and damaged organelles for lysosomal degradation, plays crucial roles in survival and diseased conditions. Efforts were made to understand the role of autophagy in motor neuron degeneration and to target autophagy in motor neuron for ALS treatment. However, results were quite contradictory. Possible autophagy defects in other cell types may also complicate the results. Here, we examined autophagy activity in skeletal muscle of an ALS mouse model G93A. Through overexpression of a fluorescent protein LC3‐RFP, we found a basal increase in autophagosome formation in G93A muscle during disease progression when the mice were on a regular diet. As expected, an autophagy induction procedure (starvation plus colchicine) enhanced autophagy flux in skeletal muscle of normal mice. However, in response to the same autophagy induction procedure, G93A muscle showed significant reduction in the autophagy flux. Immunoblot analysis revealed that increased cleaved caspase‐3 associated with apoptosis was linked to the cleavage of several key proteins involved in autophagy, including Beclin‐1, which is an essential molecule connecting autophagy and apoptosis pathways. Taking together, we provide the evidence that the cytoprotective autophagy pathway is suppressed in G93A skeletal muscle and this suppression may link to the enhanced apoptosis during ALS progression. The abnormal autophagy activity in skeletal muscle likely contributes muscle degeneration and disease progression in ALS.

## Abstract (precis)

Amyotrophic lateral sclerosis (ALS) is a fetal neuromuscular disease, in which skeletal muscle is substantially affected. This study discovered a specific defect in autophagy pathway in skeletal muscle, which likely plays an important role in ALS disease progression.

## Introduction

ALS is a fatal neuromuscular disease characterized by the progressive loss of motor neuron and skeletal muscle atrophy. Currently, there is no effective treatment. Ninety percent cases of ALS are sporadic (SALS), with about 10% being familial (FALS) (Pasinelli and Brown 2006). Both SALS and FALS manifest similar pathological and clinical phenotypes, suggesting that different initiating molecular insults promote a similar neurodegenerative process. Mutations in the Cu/Zn‐superoxide dismutase gene (SOD1) are associated with a fraction of FALS (Pasinelli and Brown 2006). Transgenic mouse model harboring human ALS‐causing SOD1 mutations (i.e., G93A) recapitulates the neuronal and muscle impairment of human ALS patients and thus has been widely used by ALS research community (Gurney et al. 1994) and was used in this study.

Studies on ALS mouse models and patients show that accumulation of abnormal protein inclusions is involved in motor neuron degeneration (Nassif and Hetz 2011). Autophagy is a tightly regulated intracellular process that targets misfolded proteins and damaged organelles for lysosomal degradation. It also plays crucial roles in survival and diseased conditions (Mizushima 2007). Dysregulation of autophagy occurs in various neurodegenerative diseases (Banerjee et al. 2010). Upregulated autophagy activity has been reported in the spinal cord of ALS patients and animal models (Morimoto et al. 2007; Li et al. 2008; Sasaki 2011; Zhang et al. 2011). However, contradictory results were also reported. For example, there was no detected increase in the expression of autophagosome marker LC3‐II in the spinal cord of ALS mouse model G93A (Crippa et al. 2013). Some studies suggest that agents that activate autophagy would help remove misfolded proteins in the motor neuron and limit ALS progression (Hetz et al. 2009; Ikenaka et al. 2013). One example is Rapamycin (an mTOR inhibitor), which is known to alleviate disease symptoms in other neurodegenerative diseases (i.e., Alzheimer's and Huntington's diseases) (Bove et al. 2011). However, the result of Rapamycin treatment in ALS is rather complicated (Nassif and Hetz 2011; Chen et al. 2012). Rapamycin alleviated disease progression in ALS TDP‐43 mice (Wang et al. 2012), but augmented motor neuron degeneration in ALS mouse models with SOD1 mutations (Zhang et al. 2011), with an adverse effect on muscle performance (Bhattacharya et al. 2012). Contradictory results were also obtained when lithium was used to activate autophagy to treat ALS mouse models (Fornai et al. 2008; Pizzasegola et al. 2009). The mixed results from promoting autophagy in ALS mouse models could result from many different reasons. One could be abnormal autophagy activity in nonmotor neuronal cells that may affect whole‐body homeostasis and complicate the therapeutic situation, since other cell types may also contribute to ALS progression (Boillee et al. 2006). Thus, more mechanistic studies are needed to further understand the dysregulation of autophagy not only in the motor neuron but also in other cell types in the context of ALS in order to effectively target autophagy for treating this devastating disease.

The degeneration of motor neuron limits neuron‐to‐muscle signaling and leads to severe muscle atrophy in ALS, while the retrograde signaling from muscle‐to‐neuron, which is important for axonal growth and neuromuscular junction maintenance (Nguyen et al. 2000), is also lost in ALS progression. Indeed, accumulating evidence supports that muscle plays an active role in ALS progression (Gonzalez de Aguilar et al. 2008; Luo et al. 2013). Expression of muscle‐specific IGF‐1 in G93A mice was shown to enhance motor neuronal survival, delaying the disease onset and progression (Dobrowolny et al. 2005). Our recent studies have shown that defects in Ca signaling affect neuromuscular junction remodeling and contribute to muscle atrophy during ALS progression (Zhou et al. 2010; Yi et al. 2011; Luo et al. 2013). In further support of an active role of muscle in ALS progression, published studies have shown that transgenic mouse with muscle‐specific overexpression of SOD1 mutation (G93A) developed age‐related neurologic and pathologic phenotypes consistent with ALS [(Wong and Martin 2010), also see (Dobrowolny et al. 2008)]. Skeletal muscle comprises around 40% of whole‐body lean mass (Neel et al. 2013) and is substantially affected in ALS. Increasing evidence demonstrates that skeletal muscle autophagy plays an important role in control body nutrient trafficking and metabolism, which is critical for whole‐body metabolic homeostasis (Neel et al. 2013). Moreover, recent studies have found autophagy dysregulation in muscular dystrophies (Grumati et al. 2010; Pauly et al. 2012). However, it is not known whether there are defects in autophagy activity in ALS skeletal muscle.

In this study, we explored the autophagy activity in skeletal muscle of an ALS mouse model G93A at different disease stages. A remarkable discover from this study is that autophagy flux in skeletal muscle is significantly suppressed during ALS progression. Following a well‐established autophagy induction procedure, the skeletal muscle of normal control mice showed increased autophagy flux as expected (Mammucari et al. 2007; Ju et al. 2010), whereas the G93A skeletal muscle lost the capacity to activate the autophagy pathway in response to the same autophagy induction procedure. Further mechanistic studies identified apoptosis‐related molecules that were likely associated with this suppressed autophagy flux in G93A skeletal muscle. There may be a potential abnormal crosstalk between autophagy and apoptosis pathways in G93A skeletal muscle that constitutes pathological sequences of skeletal muscle degeneration in the course of ALS progression.

## Materials and Methods

### Gene transfection in skeletal muscle of adult mice

ALS model mice (G93A) (Gurney et al. 1994) and the age‐matched normal mice (wild type) as the control were used in the study. Transfection was done using an electroporation protocol modified from Pouvreau et al. (2007) and Yi et al. (2011). Briefly, anesthetized mice were injected with 10 *μ*L of 2 mg/mL hyaluronidase dissolved in sterile saline at the ventral side of the hind paws using a 29‐gauge needle. One hour later, 5–10 *μ*g plasmid DNA (LC3‐RFP, Origene, Rockville, MD, Cat# RC100052) in 10 *μ*L sterile saline was injected at the same site. Fifteen minutes later, two electrodes (gold‐plated stainless steel acupuncture needles) ~9 mm apart were placed at the starting lines separating paw and toes. Twenty pulses of 100 V/cm at 20 ms/pulse were applied at 1 Hz (ECM 830 Electro Square Porator, BTX, Holliston, MA). Seven days later, the animal was euthanized by CO_2_ inhalation and the flexor digitorum brevis (FDB) muscles were removed for functional studies. All experiments were carried out in strict accordance with the recommendation in the Guide for the Care and Use of Laboratory Animals of the National Institutes of Health. All experimental protocols were approved by the IACUC of Rush University.

### Muscle fiber preparation

Individual muscle fibers were isolated following a protocol described previously (Zhou et al. 2010; Yi et al. 2011). FDB muscles were digested in modified Krebs solution (0 Ca^2+^) plus 0.2% type I collagenase for 55 min at 37°C. Following this collagenase treatment, muscle fibers were stored in an enzyme‐free Krebs solution at 4°C, and used for imaging studies within 12 h in the same day.

### Fluorescent dye loading and confocal microscopic imaging

**Figure 1.**
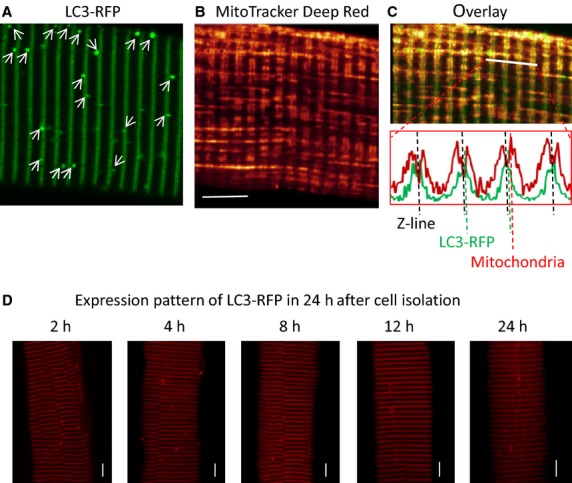
Characterization of LC3‐RFP expression in FDB muscle fibers. A live FDB muscle fiber expressing LC3‐RFP (A) was labeled with MitoTracker Deep Red (B) to obtain simultaneously recorded images. LC3‐RFP forms double rows in the FDB muscle fiber. (C) The overlay image of (A) and (B) (the top panel) and the corresponding fluorescence profiles (the lower panel) are used to identify the targeting of LC3‐RFP. The red dashed line marks the peak fluorescence of mitochondria. It is well known that mitochondria form double rows along both sides of the *Z*‐line. Thus, the *Z*‐line can be defined in the middle of the peak fluorescence of mitochondria and is marked by the black dashed line in the profile. The green dash line marks the peak fluorescence of LC3‐RFP. The alignment of those three dashed lines indicates that LC3‐RFP protein molecules are evenly distributed closely to the Z‐line before forming autophagosomes. The LC3‐RFP fluorescent puncta formed outside the Z‐line indicated by white arrows in (A) are considered as autophagosomes. (D) Representative images of muscle fibers expressing LC3‐RFP taken at different time points during 24 h after the fiber isolation. Note there are no time‐dependent changes in the expression pattern of LC3‐RFP. Bar: 10 *μ*m.

**Figure 2.**
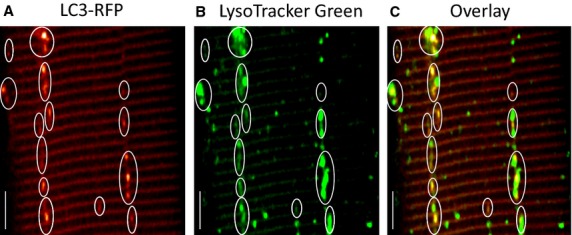
LC3‐RFP fluorescent vesicles are always in close contact with lysosomes. Muscle fibers expressing LC3‐RFP were labeled by LysoTracker Green to simultaneously monitor LC3‐RFP fluorescent puncta and lysosomes. (A) FDB fiber expressing LC3‐RFP. (B) Lysosomes in the same FDB fiber were labeled with LysoTracker Green. Note that LC3‐RFP fluorescent puncta are always in close contact with lysosomes (C), indicating that LC3‐RFP fluorescent puncta are autophagosomes. Bar: 10 *μ*m.

**Figure 3.**
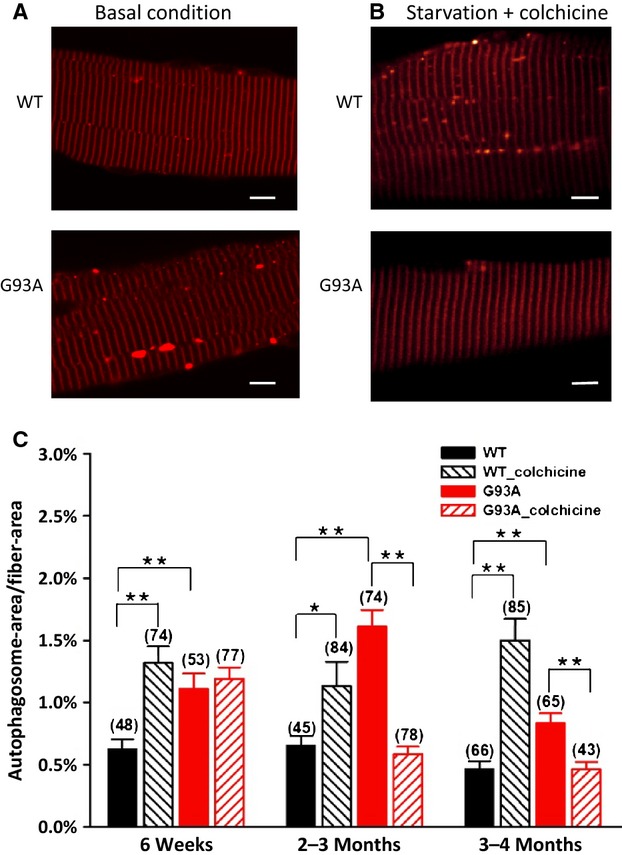
Quantification of autophagosome formation in muscle fibers under basal and autophagy induction conditions. Both G93A and control (WT) FDB muscle were transfected with LC3‐RFP. (A) Representative images of G93A and WT FDB muscle fibers derived from 2‐month‐old mice under the basal condition, note more LC3‐RFP puncta (autophagosomes) in the G93A fiber. (B) Representative images under autophagy induction condition (starvation plus colchicine), note reduced autophagosome formation in the G93A fiber. (C) Quantification of autophagosome in three age groups under both conditions. G93A muscle fibers show increased autophagosome formation in all age groups under the basal condition. However, following the autophagy induction procedure, control muscle fibers (WT_colchicine) show enhanced autophagosome formation as expected, while G93A muscle fibers from 6‐week‐old mice (G93A_colchicine) show no further increase in autophagosome formation, and muscle fibers derived from G93A mice older than 2 months show a dramatic reduction in autophagosome formation. These data suggest that there is a suppression of autophagy flux in G93A skeletal muscle during ALS progression. The number on the top of each bar represents the number of FDB fibers included in each group, 3–5 mice/group, *P < 0.05, **P < 0.01. Bar: 10 *μ*m.

**Figure 4.**
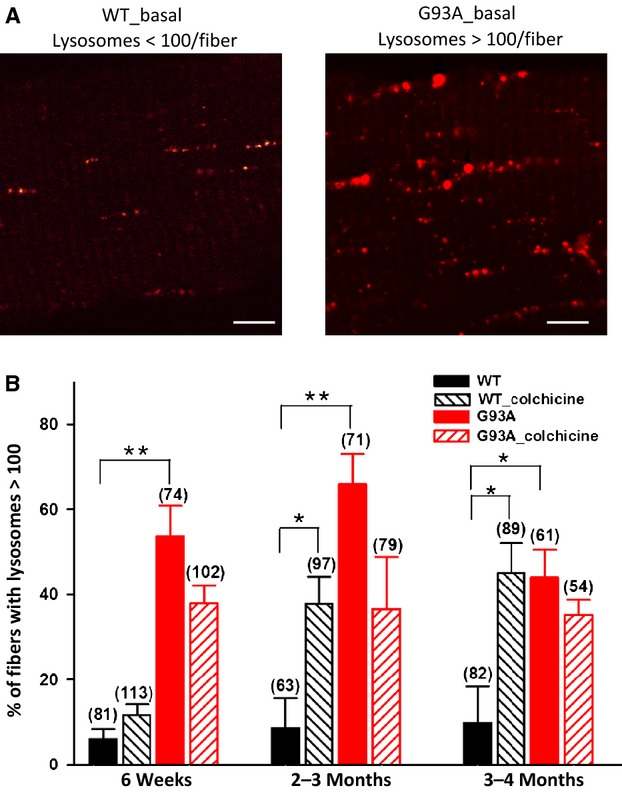
Lysosome formation in muscle fibers under basal and autophagy induction conditions. Both G93A and control (WT) FDB muscle fibers were labeled with LysoTracker Green. Under normal diet (basal condition), the majority of individual WT muscle fibers contains <100 lysosomes/fiber, while the majority of G93A fibers contains lysosomes more than 100/fiber. (A) Representative images of individual WT and G93A FDB muscle fibers under basal condition. (B) The percentage of fibers containing lysosomes more than 100/fiber was quantified for each group. Note, under basal condition, G93A muscle fibers have shown significant more numbers of lysosomes/fiber at all age groups. WT muscle fiber (older than 2 months) responded to the autophagy induction procedure (WT_colchicine) with dramatically increased formation of lysosomes. However, G93A muscle fibers (G93A_colchicine) showed no further increase in lysosomes formation following the same procedure. The number on the top of each bar represents the number of FDB fibers included in each group, 3‐5 mice/group, *P < 0.05, **P < 0.01. Bar: 10 *μ*m.

FDB muscle fibers were incubated with 500 nmol/L MitoTracker Deep Red for 10 min at 25°C to visualize mitochondria, and 500 nmol/L LysoTracker Green 15 min to visualize lysosomes. In some cases, images of MitoTracker Deep Red or LysoTracker Green signals were simultaneously recorded with LC3‐RFP. LysoTracker Green was excited at 488 nm and its emitted fluorescence was collected at 498–540. MitoTracker Deep Red was excited at 633 nm and emitted at 647–700, while LC3‐RFP was excited at 543 and its emitted fluorescence was collected at 560–620. A confocal microscope (SP2‐AOBS with a 40× and 1.2 NA water‐immersion objective, Leica Microsystem, Buffalo Grove, IL) was used. Both MitoTracker Red and LysoTracker Green were purchased from Invitrogen (Grand Island, NY). There are two different sets of confocal scanning settings used for the live cell image acquisition. The images in Fig. 1A and B were acquired at a scanning setting with a high zoom and 16‐line averages. This scanning setting provides a high‐resolution image to distinguish the Z‐line in live muscle fibers, which allowed us to obtain the profiles of mitochondria and LC3‐RFP striated pattern along the Z‐line (Fig. 1C). However, this scanning setting is not suitable for the quantification of autophagosomes or imaging of lysosomes, because it causes potential laser damage at the local fiber area and it is a time‐consuming process. The images taken for demonstrating the time‐dependent expression of LC3‐RFP (Fig. 1D), analysis of autophagosome formation and lysosomes (Figs 2–4) were done under a scanning setting with a lower zoom and two‐line averages, which limited the laser damage and potential biases for the data analysis.

### Pharmacological reagents and the autophagy induction protocol

An established autophagy induction procedure (Ju et al. 2010) was applied to examine autophagy flux in skeletal muscle. Colchicine was applied by intraperitoneal injection at a dose of 0.4 mg/kg/day for 4 days. On the 3rd day, the mice were put into a new cage and supplied only with water to initiate a 36‐h starvation period. After this period skeletal muscles were removed for examination of autophagosome formation and immunoblot analysis. Colchicine was dissolved in deionized water at a concentration of 4 mg/mL. Then, 20‐*μ*L colchicine (80 *μ*g) solution was dissolved in 800 *μ*L saline for intraperitoneal injection. Colchicine and other chemicals were obtained from Sigma.

### Immunoblot assay

Tibialis anterior muscles from normal and G93A mice were collected and homogenized in protein extraction buffer containing protease inhibitor cocktail (Thermo Scientific, Waltham, MA) using motorized homogenizer (Wheaton, Millville, NJ). Protein concentrations were determined by BCA protein assay (Thermo Scientific). Equal mass protein samples (30 *μ*g) were subjected to SDS‐polyacrylamide gel electrophoresis before transferred to nitrocellulose (Bio‐Rad, Hercules, CA), and immunoblotted with primary antibodies. The antibodies used were as follows: anti‐phospho‐mTOR (ser2448) (Cell Signaling, Danvers, MA, 5536), anti‐mTOR (Cell Signaling, 2983), anti‐lysozyme (Santa Cruz, Dallas, TX, sc‐27958), anti‐caspase‐3 (Cell Signaling, 9665), anti‐Beclin 1 (Santa Cruz, sc‐10086), anti‐Bcl‐xl (Santa Cruz, sc‐8392). All these antibodies were used at a 1:1000 dilution. One antibody, anti‐tubulin (Santa Cruz Biotechnology), was used at a 1:10000 dilution. Results were visualized with ECL reagents (Thermo Scientific). Densitometry evaluation was conducted using ImageJ software (NIH, Bethesda, MD).

### Statistical analysis and image processing

IDL 7.0 (IDL, ITT Visual Information Solutions, Boulder, CO) was used for image processing. Sigmaplot 11.0 (San Jose, CA) and Microsoft Excel (Redmond, WA) were used for data analysis. Results are represented as mean ± SEM with statistical significance determined by Student's *t*‐test.

## Results

### Characterization of autophagosome formation in live skeletal muscle fibers

The microtubule‐associated protein light chain 3 (LC3) is one of the major protein markers of autophagosome in eukaryotes (Kabeya et al. 2000) and fluorescent LC3 fusion proteins (LC3‐GFP or LC3‐RFP) have been successfully used to monitor autophagosome formation in various types of cells (Mizushima et al. 2004; Mammucari et al. 2007). Here, we used LC3‐RFP to follow the autophagosome formation in live ALS skeletal muscle fibers. We first characterized the expression pattern of LC3‐RFP protein in skeletal muscle of normal mice in our experimental condition. The LC3‐RFP plasmid was transfected into the FDB muscle of a live mouse. Seven days post transfection, the transfected FDB muscle was collected and enzyme‐digested to isolate individual muscle fibers for confocal imaging study. Unlike reported for other cells, the cytosol expression of LC3‐RFP in skeletal muscle fibers was not homogeneous but instead it formed transversal parallel rows (Fig. 1A). Because it is known that mitochondria localize to double rows along the Z‐line in FDB muscle (Boncompagni et al. 2009; Zhou et al. 2010), we used mitochondria as a standard marker to define the localization of LC3‐RFP inside the muscle fiber. The muscle fibers expressing LC3‐RFP were incubated with MitoTracker Deep Red (MitoTracker) to visualize mitochondria (Fig. 1B). The overlay image of LC3‐RFP and MitoTracker suggests that LC3‐RFP is distributed evenly along the Z‐line between the double rows of mitochondria (Fig. 1C). The autophagosome formation is indicated by puncta of intense LC3‐RFP fluorescence as marked by white arrows in Fig. 1A. The size and shape of LC3‐RFP puncta are random in both WT and G93A muscle fibers, while the evenly striated distribution of LC3‐RFP along the Z line is the same in both WT and G93A muscle fibers. In addition, we examined the expression pattern of LC3‐RFP in muscle fibers in the time period of 24 h following the collagenase treatment when muscle fibers were kept at 4°C. Figure 1D demonstrates that there are no time‐dependent changes in the expression pattern of LC3‐RFP.

Autophagy is a cellular process that targets autophagosomes for lysosomal degradation. To verify if LC3‐RFP puncta are indeed autophagosomes, we compared the localization of LC3‐RFP puncta and lysosomes. The isolated live FDB muscle fibers expressing LC3‐RFP (Fig. 2A) were incubated with LysoTracker Green to visualize lysosomes. The LC3‐RFP and LysoTracker Green were then excited at 543 nm and 488 nm, respectively. Interleaved excitation and spectrally separated emission wavelengths permitted simultaneous recording of the LC3‐RFP and LysoTracker Green signals (Fig. 2). Lysosomes are observed as green fluorescent puncta in Fig. 2B. The overlay image of LC3‐RFP and LysoTracker Green (Fig. 2C) indicates that the majority of LC3‐RFP fluorescent puncta are in close contact with lysosomes, suggesting that the sites of concentrated LC3‐RFP puncta are indeed autophagosomes.

### Autophagosome formation in G93A skeletal muscle is elevated at the basal level, but reduced following an autophagy induction procedure

To evaluate the autophagy activity in ALS skeletal muscle during the disease progression, we transfected LC3‐RFP plasmid into the FDB muscle of G93A mice at different disease stages: the earlier stage [6 weeks, asymptomatic stage without axonal withdrawal reported (Frey et al. 2000)], 2–3 months [still asymptomatic stage but axons have begun to pull away from muscle fibers], and 3–4 months [disease onset and ALS‐like phenotype becomes well established (Gurney et al. 1994)]. Parallel experiments were also conducted using the FDB muscle of age‐matched wild‐type control mice. Autophagosome formation was first examined in G93A and wild‐type muscles at basal condition, in which mice were kept on a regular diet without inducing autophagy activity. Representative images from 2‐month‐old G93A and wild‐type muscle are shown in Fig. 3A. To quantify the autophagosome formation, the ratio of the total area of autophagosomes and the area of the imaged fiber segment was determined. The area of individual autophagosome punctum (see Fig. 1A) was calculated by selecting the region inside the punctum with the fluorescence intensity 10% above the background. As demonstrated in Fig. 3C (red bar), the autophagosome formation at the basal level was increased in G93A skeletal muscle at all tested ages compared to the wild‐type muscle.

It is well known that an apparent increase in autophagosome formation is not a necessary indication of an activation of the autophagy pathway or autophagy flux, because it could reflect either induction of autophagy or reduced clearance of autophagosomes (Mammucari et al. 2007; Klionsky et al. 2008; Ju et al. 2010). Thus, autophagy flux has been used to evaluate the complete process of autophagy (Klionsky et al. 2008; Ju et al. 2010). Autophagosomes are trafficked along microtubules to fuse with lysosomes to form autolysosomes (Banerjee et al. 2010). To evaluate the autophagy flux in the G93A skeletal muscle, we treated the mice with colchicine, a microtubule depolymerizing agent, to block the fusion of autophagosomes with lysosomes (Ju et al. 2010). In the meanwhile, a starvation procedure was applied to activate the autophagy pathway. This autophagy induction procedure was well established previously to evaluate autophagy flux in skeletal muscle (Ju et al. 2010). In the presence of colchicine, increased autophagosome formation induced by starvation should suggest an increased autophagy flux (Ju et al. 2010). Mice were injected with colchicine for 4 days. On the 3rd day, the mice were provided with water only to initiate a 36‐h starvation period in order to activate the autophagy pathway (Fig. 3B). As expected, the autophagy induction procedure promoted autophagosome formation in skeletal muscle of normal mice at all ages (Fig. 3C, black striped bar). However, the same autophagy induction procedure did not promote further autophagosome formation in young G93A mice, and even reduced autophagosome formation in G93A mice at the ages older than 2 months (Fig. 3C, red striped bar). Thus, basal autophagosome formation is elevated in ALS skeletal muscle, but starvation no longer evokes additional activity, indicating a suppressed autophagy flux in G93A skeletal muscle. This suggests that the reserved capacity to form autophagosome (i.e., increase autophagy flux) in a stressed condition may be lost in the skeletal muscle of G93A mouse model upon the disease progression.

### Altered lysosome activity in G93A skeletal muscle

Autophagosomes are targeted for lysosomal degradation. Thus, we examined the lysosomal activity in G93A skeletal muscle. Lysosomes were visualized by incubated muscle fibers with LysoTracker Green (see Fig. 2). Lysosome activity was evaluated as the amount of lysosome vesicles contained in individual muscle fibers. We counted the percentage of fibers with lysosomes more than 100/fiber for each group. Representative images of G93A and WT muscle under the basal condition are shown in Fig. 4A. The comparison was done in age‐matched wild‐type and G93A muscle fibers before and after the autophagy induction procedure. The percentage of muscle fibers containing >100 lysosomes/fiber is summarized in Fig. 4B. Under normal diet (basal condition), fewer than 10% of wild‐type fibers had >100 lysosome vesicles in all age groups. This indicates that there is relatively quiescent lysosomal activity in wild‐type muscle at the condition without autophagy induction (Fig. 4B, black bar). Following the autophagy induction procedure, the number of lysosomes was significantly increased in wild‐type muscle (Fig. 4B, black striped bar). In the absence of starvation, G93A muscle derived from all age groups showed significant increase in the number of lysosomes comparing to the control (Fig. 4B, red bar), indicating an enhanced lysosome activity in G93A skeletal muscle at the basal level. However, the starvation procedure no longer evoked more lysosome activity in G93A muscle derived from all age groups (Fig. 4B, red striped bar). The data suggest that the lysosome activity is altered in G93A muscle and the lack of response of lysosomal activity to the autophagy induction procedure was in line with the reduced autophagy flux in G93A muscle in ALS progression.

### Interplay between autophagy and apoptosis pathways in G93A skeletal muscle

**Figure 5.**
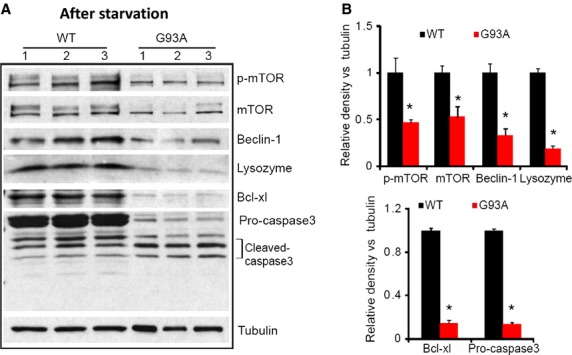
Immunoblot analysis of key proteins involved in both autophagy and apoptosis pathways in G93A and control skeletal muscle following the same autophagy induction procedure. (A) G93A muscles show pronounced reduction in the amount of proteins related to autophagy pathway: p‐mTOR, mTOR, Beclin‐1 and Lysozyme. G93A muscles also show activated apoptosis with pronounced decrease in proapoptotic protein Bcl‐xl and the increase in cleaved caspase‐3. (B) The relative intensity of the protein bands to tubulin. Relative protein band intensity was analyzed using ImageJ (NIH) (*n* = 3 mice for control and G93A, respectively, *P < 0.05).

Autophagy flux is a dynamic, multistep process that can be modulated at several steps (Klionsky et al. 2008). To further understand the molecular basis of the suppressed autophagy flux in G93A skeletal muscle, we conducted immunoblot analysis to determine if other autophagy‐related proteins and pathologic changes might be involved. As the suppressed autophagosome flux became more evident when G93A mice reached the age of 2–3 months, the skeletal muscles used for immunoblot analysis were taken from 3‐month‐old G93A and age‐matched wild‐type mice following the same autophagy induction procedure. As illustrated in Fig. 5A, the expression of the upstream proteins [mTOR, p‐mTOR (phosphorylated‐mTOR), Beclin‐1] and the downstream protein (lysozyme) (Marino et al. 2014) in the autophagy pathway were significantly reduced in G93A muscle, although there was no significant change in the ratio of p‐mTOR/mTOR comparing to the wild‐type muscle. This suggests that autophagy‐related proteins are likely exhausted during ALS progression.

Autophagy is a programmed survival strategy, whereas apoptosis is a process of programmed cell death. Accumulating evidence suggests that there is an interplay between these two intracellular pathways (Maiuri et al. 2010; Marino et al. 2014; Mukhopadhyay et al. 2014). Beclin‐1 is not only an essential molecule involved in the autophagy pathway but is also known to play a critical role in connecting autophagy and apoptosis activities (Djavaheri‐Mergny et al. 2010). Expression of Beclin‐1 is dramatically reduced in G93A muscle under the stressed condition (Fig. 5A). This finding led us to examine the apoptosis pathway in G93A skeletal muscle by evaluating the expression of well‐established apoptotic markers, Bcl‐xl and caspase‐3. These two proteins have also been shown to play a role in regulating the autophagy pathway (Djavaheri‐Mergny et al. 2010; Mukhopadhyay et al. 2014). As shown in Fig. 5B, the expression of antiapoptotic protein Bcl‐xl was dramatically decreased in G93A muscle. In addition, G93A muscle had a higher ratio of cleaved caspase‐3 to pro‐caspase‐3 (Fig. 5B). The significant increase in the cleavage of caspase‐3 indicates enhanced apoptosis in G93A skeletal muscle under the autophagy induction condition. Our data indicate that the suppressed autophagy flux in G93A muscle fibers is accompanied by enhanced apoptosis, suggesting that there may be a potential interplay between these two intracellular pathways during ALS progression.

## Discussion

Our current study has revealed abnormal autophagy activity in skeletal muscle of an ALS mouse model G93A. Autophagy flux is significantly suppressed in G93A skeletal muscle during disease progression. This abnormality begins early and becomes severe at later stages. The reduced autophagy flux is likely due to the reduced expression level of key molecules involved in autophagy. We have also identified a potential crosstalk between autophagy and apoptosis pathways in G93A skeletal muscle that likely promotes muscle atrophy and disease progression.

Overexpression of LC3‐fluorescent autophagy marker protein does not affect the endogenous autophagy process (Mizushima et al. 2004). To assess the autophagy activity in live skeletal muscle cells, we have overexpressed LC3‐RFP in skeletal muscle of live mice. In nonmuscle cells the LC3‐fluorescent protein distributes homogeneously inside cytosol. In skeletal muscle cells, expression of LC3‐fluorescent proteins shows a striated pattern, but the intracellular trafficking of LC3 protein has not been characterized (Mizushima et al. 2004; Mammucari et al. 2007). Here, we characterized the targeting of LC3 in skeletal muscle fibers by costaining live muscle fibers expressing LC3‐RFP with the mitochondrial and lysosome markers. We found that the LC3 protein evenly distributes along the Z‐line before forming vesicle‐like (puncta) autophagosomes (Figs 1 and 3).

Using LC3‐RFP as an autophagosome marker, we explored autophagosome formation in skeletal muscle of G93A mice at different disease stages. On a regular diet, G93A skeletal muscle had increased autophagosome formation indicated by increased number of LC3‐RFP fluorescent puncta at all tested ages. This result is consistent with the biochemical study of Crippa et al. (2013), where they reported enhanced expression of LC3‐II in skeletal muscle of G93A mice at the age of 2 and 4 months. However, it is not clear if an elevated level of LC3‐II or increased number of LC3‐RFP marked autophagosomes is a true indication of autophagy activation in G93A skeletal muscle (Klionsky et al. 2008). We thus investigated autophagy flux in G93A muscle by blocking the fusion of autophagosomes with lysosomes while autophagy was promoted by a starvation procedure that is well established to evaluate autophagy flux in mammalian skeletal muscle (Mammucari et al. 2007; Ju et al. 2010). We found that autophagy flux in G93A muscle was suppressed. Although there was an increase in autophagosome formation under basal nonstarvation condition, young G93A mice did not have the capacity to form more autophagosomes when challenged by starvation. In older G93A mice (>2 months), there was a significant reduction in autophagosome formation in skeletal muscle following this autophagy induction procedure. In contrast, the age‐matched wild‐type mice responded to the starvation with a robust increase in the number of autophagosome puncta as expected (Mammucari et al. 2007; Ju et al. 2010) (Fig. 3). Thus, our data demonstrated that autophagy flux in G93A skeletal muscle was significantly suppressed. In line with the result from autophagy studies, the G93A muscle also had enhanced basal lysosomal activity, but lacked the ability to produce more lysosomes when challenged by starvation.

Two pathological events occur in skeletal muscle during ALS progression. One is the axonal withdrawal of motor neuron from the neuromuscular junction, leading to skeletal muscle denervation. The other is the expression of the toxic mutant SOD1 protein in skeletal muscle. It has been shown that denervation enhances autophagy activity in skeletal muscle (Romanello et al. 2010; O'Leary et al. 2012). The enhanced autophagy activity in G93A skeletal muscle at the basal condition could be a consequence of motor axonal withdrawal during ALS progression. However, the early increase in autophagy activity found in young G93A mice at 6 weeks may have other reasons, because there is no detectable axonal withdrawal at this age (Frey et al. 2000). Romanello et al. (2010) showed that increased mitochondrial fission activity promoted autophagosome formation in skeletal muscle. We also reported that increased fission activity in G93A muscle at young age was induced directly by mutant SOD1^G93A^ protein in the absence of axonal withdrawal (Luo et al. 2013). Thus, the mutant SOD1^G93A^ protein may also play a role in promoting the basal autophagy activity in G93A muscle, especially at early stages. Indeed, overexpression of mutant SOD1 in cultured muscle cell line C2C12 promotes autophagy activation (Onesto et al. 2011). It seems that both pathological events listed above activate autophagy in G93A muscle at the basal condition. This is likely a cytoprotective response to help remove misfolded proteins and damaged organelles. However, constantly chronic enhancement of basal autophagosome formation could end up exhausting the intracellular machinery related to autophagy pathway. This could explain why G93A skeletal muscle loses the capacity to further increase autophagy flux when challenged by starvation.

We further investigated the potential molecular mechanism underlying the suppressed autophagy flux in G93A skeletal muscle. Immunoblot analysis of G93A skeletal muscle was applied to examine protein expression of other key molecules (mTOR, Beclin‐1 and lysozyme) involved in the autophagy pathway following the same autophagy induction procedure. We found that expression of these autophagy‐related molecules was dramatically reduced in G93A muscle. This is consistent with the reduced autophagosome formation observed by overexpression of LC3‐RFP in live G93A skeletal muscle following the same autophagy induction procedure. mTOR is a negative regulator of autophagy activity in the upstream of the autophagy pathway. In a nutrient‐rich environment, mTOR is activated by phosphorylation to suppress autophagy. In a nutrient‐depleted environment, phosphorylation of mTOR is inhibited, leading to autophagy activation (Diaz‐Troya et al. 2008). Thus, a reduced ratio of p‐mTOR/mTOR is an indication of autophagy activation (Zhang et al. 2011). We evaluated the protein level of both p‐mTOR and mTOR in G93A muscle and found no significant changes in the ratio but a dramatic reduction in the overall mTOR expression level (including p‐ mTOR and mTOR) compared to the wild‐type muscle. Since there is reduced expression of mTOR in skeletal muscle of transgenic mice with restricted‐muscle expression of SOD1^G93A^ (Dobrowolny et al. 2011), mutant SOD1^G93A^ overexpression could be responsible for the reduced mTOR expression in G93A skeletal muscle. The transgenic ALS mouse model G93A used here systematically expresses mutant SOD1^G93A^ (Gurney et al. 1994) and our previous study also showed high expression level of SOD1^G93A^ in skeletal muscle of this mouse model (Luo et al. 2013). It is possible that expression of mutant SOD1^G93A^ is one of reasons for the reduced level of mTOR in G93A skeletal muscle, although the detailed molecular mechanism needs further investigation. Since there is no change in the p‐mTOR/mTOR ratio in G93A muscle, suggesting that other mechanisms should be considered for explaining the observed reduction in muscle autophagy flux of G93A mice. Thus, we further examined apoptosis activity in G93A muscle following the same starvation procedure.

Apoptosis is the process of programmed cell death involved in skeletal muscle degeneration in various pathophysiological conditions. Activation of apoptosis occurs in skeletal muscle following denervation (Siu and Alway 2005; Adhihetty et al. 2007). Increased apoptosis also occurs in skeletal muscle of ALS patients (Tews et al. 1997; Schoser et al. 2001) and in ALS mouse models observed as an upregulated caspase activity (Kaspar et al. 2003; Dobrowolny et al. 2011). Here, we also identified upregulated apoptosis in G93A skeletal muscle evidenced by reduced Bcl‐xl protein and enhanced cleavage of caspase‐3. Notably, the upregulated apoptosis is companied with reduced autophagy flux in G93A muscle. We speculated that there may be a crosstalk between autophagy and apoptosis in G93A muscle. The mechanisms linking autophagy and apoptosis, however, are not fully defined. Accumulating evidence has revealed that Beclin‐1 is not only essential for autophagy but also is identified as an essential component connecting autophagy and apoptosis pathways (Djavaheri‐Mergny et al. 2010; Marino et al. 2014). Studies have shown that caspase‐mediated cleavage of Beclin‐1 inactivates autophagy and enhances apoptosis (Luo and Rubinsztein 2010; Wirawan et al. 2010). We examined Beclin‐1 in G93A muscle and discovered that its expression level was dramatically reduced following the autophagy induction procedure. Thus, cleavage of Beclin‐1 initiated by apoptosis could be a critical event leading to the suppression of autophagy flux in G93A skeletal muscle.

**Figure 6.**
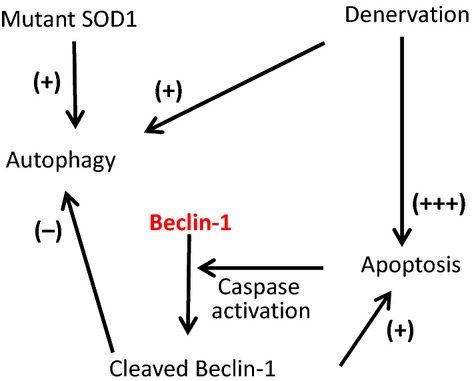
Proposed pathogenic sequences in muscle of the G93A mouse model. During ALS progression, both accumulation of mutant SOD1 protein and motor axonal withdrawal could promote muscle autophagy, a cytoprotective process. At later stage, axonal withdrawal becomes not compensable and triggers apoptosis, which leads to caspase‐mediated cleavage of autophagy‐related proteins, especially Beclin‐1. The cleavage of Beclin‐1 inhibits autophagy pathway and further promotes apoptosis. This unbalanced interplay between autophagy and apoptosis forms a vicious cycle that could promote muscle degeneration and disease progression.

Based on past and present results, one potential pathogenic sequence in G93A skeletal muscle is depicted in Fig. 6. In early ALS stages, accumulation of mutant SOD1^G93A^ inside mitochondria may play a major role in activating the autophagy pathway. As the disease progress, motor neuron withdrawal further promotes autophagy in G93A skeletal muscle. This would explain the apparent elevation in autophagosome formation in skeletal muscle observed at all ages of G93A mice maintained under normal diet. When axonal withdrawal becomes noncompensable, the severe denervation robustly promotes apoptosis pathway, particularly under stressed conditions (i.e., starvation). Apoptosis activates caspases, which lead to cleavage of autophagy‐involved proteins, including Beclin‐1. Cleaved Beclin‐1 inhibits autophagy but enhances apoptosis further (Djavaheri‐Mergny et al. 2010; Marino et al. 2014). Autophagy is considered as a cytoprotective pathway, while apoptosis is a process of programmed cell death. In the course of ALS, the motor axonal withdrawal activates apoptosis pathway and initiates caspase‐mediated cleavage of Beclin‐1, which would promote a vicious cycle of apoptosis and further suppress the autophagy pathway. This crosstalk between autophagy (decrease) and apoptosis (increase) could exacerbate skeletal muscle atrophy during ALS progression, especially at later stages or under stressed conditions.

In summary, we report here that the autophagy flux in G93A skeletal muscle is significantly suppressed during ALS progression. This suppression is likely due to the activation of apoptosis induced by motor axonal withdrawal, which results in cleavage of autophagy‐related key proteins. Among them, Beclin‐1 may play an essential role in shifting the cytoprotective autophagy response to the cell‐death apoptosis response, which exacerbates skeletal muscle atrophy in ALS. Importantly, targeting Beclin‐1 to restore the balance between autophagy and apoptosis could have great potential therapeutic value in alleviating skeletal muscle atrophy in ALS. Further investigation is required to determine the role of Beclin‐1 in motor neuron degeneration and to test if blocking the cleavage of Beclin‐1 can slow disease progression in available ALS animal models.

## Acknowledgment

We thank Dr. Michael Fill (Rush University) for valuable discussion on this project.

## Conflict of Interest

None declared.
